# From Suicidal Thoughts to Suicidal Actions: A Multidimensional Study of Entrapment, Defeat, and Negative Repetitive Thinking Through the Integrated Motivational–Volitional Model

**DOI:** 10.1192/j.eurpsy.2026.12082

**Published:** 2026-07-31

**Authors:** M. Alino Dies, D. Sanchez Reolid, M. Monferrer, M. J. Montes, A. Fernandez Caballero, P. Fernandez Sotos, J. J. Ricarte

**Affiliations:** 1 Psychiatry, Cambridgeshire and Peterborough NHS Foundation Trust, Peterborough, United Kingdom; 2 Psychiatry; 3 Computing Systems Department, University of Castilla La Mancha, Albacete; 4 Psychology, Castellon Hospital, Castellon; 5 Psychiatry, University Hospital Complex Albacete; 6 University of Castilla La Mancha, Albacete, Spain; 7 Psychology, University of Castilla La Mancha, Albacete, Spain

## Abstract

**Introduction:**

Understanding the sociodemographic and clinical profiles of patients with suicidal ideation versus suicidal behavior, and the role of cognitive–motivational factors, is key for early detection and intervention. This study examines these factors in a specialized crisis setting.

**Objectives:**

The study aimed to characterize and compare the sociodemographic and clinical profiles of patients with suicidal ideation (SI) versus suicidal behavior (SB), and to examine the influence of cognitive–motivational variables—including internal and external entrapment, defeat, and repetitive negative thinking—on both ideation and behavior. Specifically, the research sought to identify key risk factors distinguishing SI from SB, clarify how motivational variables of the IMV model manifest in a clinical outpatient sample, and explore the relationships among cognitive mechanisms to inform targeted prevention strategies.

**Methods:**

A three-phase approach was used to integrate clinical and cognitive analyses. Phase 1 described and compared sociodemographic and clinical features of 93 patients with suicidal ideation (SI) or behavior (SB). Phase 2 examined correlations among entrapment, defeat, and suicidal ideation. Phase 3 applied binary logistic regression to identify high-risk profiles based on demographic and cognitive variables.

**Results:**

Both groups shared a similar sociodemographic profile—young, single individuals with psychiatric histories—but patients with SB exhibited greater clinical vulnerability. Moderate to strong associations were observed among internal/external entrapment, defeat, and the severity of SI/SB, supporting the IMV model. Entrapment emerged as a potential mediator between defeat and suicidal ideation,. Binary logistic regression showed no significant cognitive differences between groups, reflecting the multifactorial complexity of suicide risk. Female sex and prior psychiatric emergency visits significantly distinguished SI from SB, with gender being the strongest predictor. A high-risk profile was identified in young women with repeated emergency visits and access to sedative medication, who exhibited elevated rates of SB despite no distinct cognitive profile.

**Conclusions:**

These findings support IMV-based interventions targeting entrapment, defeat, and negative repetitive thinking. Early detection, personalized follow-up, gender-sensitive approaches, and strengthened community support are recommended. The study validates the IMV model for understanding the progression from suicidal ideation to behavior and highlights the importance of precise risk profiling to guide effective, evidence-based prevention.

**Disclosure of Interest:**

None Declared

